# The Molecular Mechanisms of Fuel Utilization during Exercise

**DOI:** 10.3390/biology12111450

**Published:** 2023-11-19

**Authors:** Anna Pi, Sneha Damal Villivalam, Sona Kang

**Affiliations:** Nutritional Sciences and Toxicology Department, University of California Berkeley, Berkeley, CA 94720, USA

**Keywords:** exercise, skeletal muscle, fuel utilization

## Abstract

**Simple Summary:**

Exercise has well-known health benefits, but the way our muscles use carbohydrates and lipids as fuel during exercise is complex. It is not just about the physical activity itself; it also depends on our body’s metabolic state. The balance between using carbs and fats affects exercise performance. This review aims to provide a comprehensive look at how our bodies choose fuel sources during exercise by summarizing existing research. Understanding this can lead to advancements in exercise science and personalized exercise strategies for better health and performance.

**Abstract:**

Exercise is widely recognized for its positive impact on human health and well-being. The process of utilizing substrates in skeletal muscle during exercise is intricate and governed by complex mechanisms. Carbohydrates and lipids serve as the primary fuel sources for skeletal muscle during exercise. It is now understood that fuel selection during exercise is not solely determined by physical activity itself but is also influenced by the overall metabolic state of the body. The balance between lipid and carbohydrate utilization significantly affects exercise capacity, including endurance, fatigue, and overall performance. Therefore, comprehensively understanding the regulation of substrate utilization during exercise is of utmost importance. The aim of this review is to provide an extensive overview of the current knowledge regarding the pathways involved in the regulation of substrate utilization during exercise. By synthesizing existing research, we can gain a holistic perspective on the intricate relationship between exercise, metabolism, and fuel selection. This advanced understanding has the potential to drive advancements in the field of exercise science and contribute to the development of personalized exercise strategies for individuals looking to optimize their performance and overall health.

## 1. Introduction

Exercise is increasingly recognized as a vital component for achieving optimal health and promoting extended life expectancy. Numerous studies have proven that physical exercise has positive effects on human health and may have both preventative and mitigating effects on chronic diseases such as diabetes and cardiovascular disease [1]. The majority of our movement and contraction during exercise is attributed to our skeletal muscles [2].

Exercise can generally be classified into two main categories: endurance exercise and resistance exercise. However, it is important to note that many activities often involve a combination of both endurance and resistance components. Endurance (aerobic) exercise refers to types of physical activity performed at a lower intensity over a longer duration, such as long-distance running and swimming [3]. Resistance (strength-based) exercise refers to types of physical activity which are performed at a higher intensity over a shorter duration, such as weightlifting [3]. The benefits of exercise depend on the type of physical activity performed. Endurance training has been associated with notable improvements in cardiac output, oxygen consumption, and mitochondrial biogenesis [2]. On the other hand, resistance exercise has been demonstrated to increase muscle size, enhance neural adaptations, and improve overall strength [2]. Endurance exercise is effective in reducing cardiovascular risk factors, while resistance exercise helps maintain metabolic rate, muscle mass, and physical function with age [2]. A combination of endurance and resistance exercise has been demonstrated to induce insulin resistance and enhance glycemic control in chronic diseases like type 2 diabetes (T2D) and obesity [4,5]. The effects of exercise also depend upon duration. Acute exercise, or a single bout of exercise, results in increases in muscle protein synthesis [6] and insulin sensitivity [7]. Chronic exercise, which is exercise performed over weeks or longer, builds on the muscle-specific benefits of acute exercise and induces more peripheral insulin sensitivity [8], as well as increasing muscle mass and maximal oxygen consumption [8,9,10]. 

During exercise, skeletal muscle primarily relies on various fuel sources to meet its energy demands. Glucose, lipids, and, to a lesser extent, amino acids are the key contributors to this energy production [10]. Muscle glycogen is particularly critical during high-intensity activities as it provides an immediate source of glucose, allowing for quick bursts of energy [2,10]. Simultaneously, the liver plays a crucial role in maintaining blood glucose levels by releasing glucose into the bloodstream, ensuring a stable supply of this essential energy source [2,10].

It is important to note that lipid utilization is not limited to high-intensity activities; it extends to a broader spectrum of exercise intensities. However, during exercise, there is reduced lipolysis, the process of breaking down stored fat into fatty acids for energy, which can limit the contribution of lipids as a primary fuel source [2,10]. Nevertheless, lipid sources such as intramuscular triglycerides (IMTGs) and ketones play vital roles in energy production. IMTGs, stored within muscle cells, serve as a readily available source of energy, especially during endurance activities [2,10]. Additionally, ketone bodies, including beta-hydroxybutyrate and acetoacetate, produced by the liver, can be utilized during exercise, particularly when glucose availability is limited [2,10]. Furthermore, amino acids can serve as an energy source, which becomes particularly significant during prolonged or high-intensity exercise when glycogen and glucose stores become depleted. Amino acids can be derived from both muscle protein breakdown and circulating amino acids in the blood [2,10]. This dynamic interplay among glucose, lipids (including IMTGs and ketones), and amino acids adapts to the exercise’s intensity and duration, ensuring a continuous supply of energy to sustain muscle contractions and meet the ever-changing demands of physical activity.

The ability to smoothly transition between these fuel sources, especially glucose to lipids, is known as “metabolic flexibility” [11]. However, individuals with obesity or T2D often exhibit “metabolic inflexibility”, characterized by an impaired ability to efficiently switch between fuel sources [12]. In this review, we will delve into the significance of fuel utilization in exercise performance, focusing specifically on insights gained from genetic mouse studies.

## 2. Lipid Utilization

### 2.1. Factors Regulating Substrate Availability

Skeletal muscle expresses several fatty acid transporters, including the plasma membrane fatty acid-binding protein (FABPpm), fatty acid transport protein (FATP) 1 and 4 and fatty acid translocase CD36 (FAT/CD36) [13]. It has been shown that CD36 is critical to mediate fatty acid transport and fatty acid oxidation in skeletal muscles [14,15]. Studies by Manio et al. demonstrated that *Cd36* whole-body knockout mice exhibited a significant reduction (44%) in their ability to sustain exercise at a submaximal running speed [14,15]. This decline was attributed to the loss of CD36-mediated fatty acid transport into the muscle, leading to metabolic inflexibility—the inability to smoothly switch from glucose to fatty acid utilization during exercise. However, no changes in muscle mitochondrial enzymes and biogenesis were observed [14,15]. Studies have shown beneficial changes in whole-body metabolism in *Fatp1* whole-body knockout (KO) mice, though its specific role in exercise performance has not yet been reported [16]. *Fabp4/Fabp5* double knockout mice have also been shown to have decreased endurance exercise capacity when fasted compared to WT mice [17]. 

Low to moderate physical exercise triggers the process of lipolysis in white adipose tissue (WAT), releasing free fatty acids as a source of fuel [18]. Adipose triacylglycerol lipase (ATGL) and hormone-sensitive lipase (HSL) are the two primary enzymes responsible for triacylglycerol (TG) hydrolysis in adipose tissue. These enzymes account for approximately 95% of TG lipase activity, breaking down TG into diacylglycerol and further converting DG into monoacylglycerol [19]. Studies have demonstrated the essential role of ATGL and HSL in supplying free fatty acids during endurance exercise in mice. KO mice lacking *Atgl* or *Hsl* exhibited reduced exercise capacity. These mice failed to promote exercise-induced lipolysis and showed increased carbohydrate oxidation during exercise, leading to faster depletion of muscle and liver glycogen [19,20]. Collectively, these findings highlight the critical role of regulating free fatty acid availability for optimal endurance exercise performance.

Carnitine acts as a cofactor for enzymes such as carnitine translocase and acylcarnitine transferases I and II, which are vital for converting free long-chain fatty acids into acylcarnitines and transporting them into the mitochondrial matrix [21]. Fatty acid oxidation occurs before these substrates enter the Krebs cycle, where energy production takes place [21]. Since mitochondria membranes are impermeable to CoA esters and long-chain fatty acids, the binding of L-carnitine to acetyl groups through carnitine acyltransferase is crucial for transporting acetylated fatty acids into the mitochondria and facilitating their subsequent oxidation in the matrix [22]. A study by Kim et al. administered 150 mg/kg body weight of L-Carnitine orally to C57BL/6 wild-type mice over a three-week exercise training period. The findings revealed significant increases in mRNA and protein expression of key regulators involved in fatty acid oxidation and mitochondrial biogenesis in skeletal muscle. This enhancement in gene expression was associated with improved exercise endurance. Furthermore, the study observed a preservation of both protein and carbohydrate utilization during exercise, emphasizing the significance of optimal fuel utilization in promoting endurance exercise capacity [23].

Vascular endothelial growth factor (VEGF) plays a crucial role in promoting vasculogenesis and angiogenesis, thereby increasing blood supply to various tissues [24]. In the context of adipose tissue, VEGF facilitates the delivery of nutrients and oxygen to adipose tissue and enables the transportation of fatty acids to other parts of the body as energy substrates [24,25]. Elevated levels of circulating VEGF have been observed in obese individuals, prompting studies to investigate the role of VEGF in obesity and metabolic health through the generation of adipocyte-specific *Vegf* KO (adipo-*Vegf* KO) mice [25]. Remarkably, adipo-*Vegf* KO mice demonstrated impaired exercise tolerance, as well as hypoinsulinemia, hypoglycemia, and lower serum free fatty acid levels compared to control mice [25]. These KO mice also exhibited a significant decline of up to 40% in adipose capillarity [25]. The authors hypothesized that the reduction in capillaries compromised the transport of lipids from adipose tissue to skeletal muscle, thereby impairing the availability of fuel for endurance exercise and ultimately leading to decreased exercise endurance in the adipo-*Vegf* KO mice [25]. This finding supports the notion that the bioavailability of free fatty acids from adipose tissue is crucial for supporting optimal endurance exercise performance.

### 2.2. Factors Regulating Mitochondrial Biology

During muscle contraction, a significant portion of the required ATP is generated by mitochondria through a process called oxidative phosphorylation [26]. The effects of exercise on mitochondrial dynamics and functions vary depending on the intensity and type of exercise, often involving the regulation of proteins associated with mitochondrial pathways. Exercise can stimulate the upregulation of mitochondrial biogenesis and influence the morphology of mitochondria through fusion and fission processes, ultimately impacting mitochondrial oxidative capacity [27].

Optic atrophy 1 (OPA1) is a dynamin family member found in the inner mitochondrial membrane which regulates mitochondrial fusion [28]. It also plays a role in mitochondrial biogenesis by facilitating fusion of inner mitochondrial membranes and formation of cristae [29], particularly during physiological stresses like endurance training [30]. Surprisingly, *Opa1* haplodeficient mice exhibited reduced mitochondrial mass, lower activity of citrate synthase and cytochrome oxidase, and abnormal cristae morphology [30]. However, these mice demonstrated significantly improved endurance capacity after training, without changes in training capacity. This enhancement may be attributed to compensatory mechanisms such as increased levels of carnitine palmitoyltransferase 2, an enzyme essential for fatty acid oxidation, leading to enhanced fatty acid utilization [30]. Nuclear factor erythroid 2-related factor 2 (NRF2) is a transcription factor involved in cellular defense against oxidative stress and toxins by regulating target genes. Acute high-intensity interval exercise has been shown to activate NRF2 signaling in humans [31]. Nrf2 deficiency has been linked to skeletal muscle mitochondrial defects, including reduced content and impaired oxidative phosphorylation, leading to impaired exercise endurance in NRF2 KO mice [31]. Kelch-like ECH-associated protein 1 (KEAP1) is a negative regulator of NRF2, responsible for its degradation under normal conditions [32]. Muscle-specific *Keap1* KO mice exhibited improved endurance and resistance exercise capacity. These KO mice showed increased mitochondrial content, succinate dehydrogenase activity, and enhanced mobilization and oxidation of fatty acids during exercise. Metabolic profiling revealed reduced nucleoside triphosphates, including ATP production, possibly due to mitochondrial uncoupling or increased ATP consumption [32]. Another study, which used sulforaphane (SFN) as an NRF2 inducer to stimulate NRF2 signaling, also discovered that this stimulation resulted in an enhanced exercise capacity [33]. Reactive oxygen species (ROS) are known to increase in skeletal muscles during exercise. However, the exact effects of ROS are still debated since both insufficient ROS production and overproduction of ROS can impact exercise ability [34]. Biomarker measurements for oxidative stress and muscle damage decreased in mice that were pretreated with SFN. Downstream target genes of NRF2 included many antioxidant genes such as HO-1, NQO1, γ-GCS, and catalase [33]. The increased expression of NRF2 target genes in muscle suggests that the NRF2 pathway plays a vital role in fuel utilization during exercise [32] and regulating oxidative stress [33]. 

Peroxisome proliferator-activated receptor gamma coactivator 1-alpha (PGC-1a) is a co-transcription factor that plays a crucial role in regulating various metabolic pathways [35]. PGC-1a is involved in promoting fatty acid oxidation, mitochondrial function, and mitochondrial biogenesis [36]. Studies have demonstrated that whole-body KO mice lacking Ppargc1a exhibit impaired glucose homeostasis, altered hepatic energy metabolism, reduced expression of genes related to mitochondrial function, and exercise intolerance [35,37]. Similarly, muscle-specific *Pgc1a* KO mice have been shown to have a higher proportion of fast-twitch fibers and display exercise intolerance under basal conditions [38]. Conversely, muscle-specific *Pgc1a* transgenic mice exhibit a higher proportion of slow-twitch fibers, which are rich in mitochondria and more resistant to fatigue [39,40]. However, two additional independent studies using muscle-specific *Pgc1a* KO mice reported that PGC-1a is not essential for mediating voluntary exercise, although the impact of *Pgc1a* deficiency on exercise-induced mitochondrial biogenesis varied between the studies [41,42]. In humans, exercise induces a transient increase in PGC-1a mRNA levels [43,44], though there are conflicting results in how well mRNA changes reflect protein levels; one study found a significant increase in PGC-1a protein level directly after prolonged exercise [44] while two others did not [45,46]. 

SESTRINs (SESN1, 2, and 3) are stress-induced proteins that play important roles in various biological processes. It was shown that SESN2 and SESN3 levels increase in response to exercise in both mice and humans [47,48,49]. Overexpression of SESTRINs in flies has been shown to mimic many of the beneficial effects of exercise [50]. Studies using whole-body *Sesn* triple KO mice, which lack all three isoforms, have shown that these mice have decreased endurance capacity, impaired insulin sensitivity, and reduced mitochondrial biogenesis in muscle [50]. Mice that had whole-body KO of *Sesn1*, the primary isoform expressed in skeletal and cardiac muscles, demonstrated a decreased exercise capacity and a preference for glucose utilization during exercise, as indicated by a gradual increase in the respiratory exchange ratio (RER) [50], which is a method of measuring CO_2_ and O_2_ consumption to determine whether lipid or carbohydrate consumption is preferentially used [51,52]. The activation of SESN is thought to mimic the effects of exercise through two primary downstream pathways. First, SESNs can activate TORC2, which in turn activates AKT signaling. This facilitates insulin-dependent glucose and fat utilization, as well as the maintenance of muscle mass. Second, SESNs upregulate AMPK signaling, leading to increased levels of PGC-1a through both transcriptional and post-transcriptional mechanisms [50]. Overall, the activation of SESN during exercise appears to contribute to improved metabolic outcomes by regulating multiple signaling pathways involved in glucose and fat utilization, muscle mass maintenance, and mitochondrial biogenesis.

### 2.3. Transcription Factors

Peroxisome proliferator-activated receptor alpha (PPARα) plays a crucial role in regulating lipid and glucose metabolism [53]. Studies in both mice and humans have reported an increase in PPARα expression following exercise [54,55,56]. Investigations using whole-body *Ppara* KO mice have shown these mice have a reduction in basal fatty acid oxidation and impaired response to AICAR treatment, an AMPK activator [57,58]. Additionally, the KO mice exhibited lower levels of muscle palmitate oxidation at rest. Conversely, muscle-specific overexpression of PPARα resulted in elevated muscle palmitate oxidation and decreased expression of genes involved in glucose utilization, such as GLUT1 and GLUT4 [58]. The downregulation of these genes may be linked to the disruption of MEF2A/PGC-1α-dependent control of GLUT4 gene expression. However, there have been discrepancies between studies regarding the impact of *Ppara* deficiency on fatty acid oxidation and PPARα target gene expression in skeletal muscle [54,57,58]. These inconsistencies may be attributed to differences in the genetic backgrounds of the mice used. It is worth noting that while whole-body *Ppara* KO mice did not display significant changes in fatty acid oxidation or PPARα target gene expression, they did exhibit reduced exercise endurance and increased liver glycogen depletion [54]. This suggests that compensatory mechanisms, potentially involving high levels of PPARδ in skeletal muscle, may partially offset the loss of PPARα function. Further investigation using muscle-specific conditional KO mice will be essential in clarifying PPARα’s precise role in fuel utilization during exercise. In summary, the studies indicate that PPARα plays a vital role in exercise performance by promoting lipid utilization over glucose through the enhancement of fatty acid oxidation in skeletal muscle. Its activation during exercise may contribute to the metabolic adaptations required for efficient energy utilization during physical activity.

KLF15 is a transcription factor highly expressed in metabolic tissues and plays a crucial role in regulating macronutrient metabolism [59]. It is upregulated in skeletal muscle after exercise and particularly enriched in oxidative soleus muscle [60]. Whole-body *Klf15* KO mice exhibit reduced exercise endurance and impaired metabolic gene expression related to fatty acid utilization and mitochondrial function [60]. The skeletal muscle of KO mice shows mitochondrial defects and altered fuel utilization during exercise, with decreased lipid oxidation and increased carbohydrate usage. However, compensatory changes in gene expression related to lipid metabolism are observed. These findings highlight the importance of KLF15 in coordinating fuel utilization in skeletal muscle during exercise.

The nuclear receptor 4A1 (NR4A1), also known as NUR77, is a transcription factor preferentially expressed in fast-twitch muscle fibers, which contain fewer mitochondria and fatigue more quickly than slow-twitch fibers [39,40,61]. Studies have shown that *Nur77* expression is robustly induced by β-adrenergic stimulation and physical exercise [61]. Muscle-specific *Nur77* transgenic mice were generated, and unexpectedly, their *Nur77* transgenic muscle had an increased abundance of slow-twitch oxidative muscle fibers and mitochondrial DNA content [62]. These muscles also exhibited enhanced oxidative metabolism, indicating increased mitochondrial activity [62]. Metabolomic analysis confirmed that *Nur77* transgenic muscle favored fatty acid oxidation over glucose oxidation, resembling the metabolic profile of fasting [62]. *Nur77* expression also improved the intrinsic respiratory capacity of isolated mitochondria, likely due to increased abundance of complex I of the electron transport chain [62]. These changes in mitochondrial metabolism translated to improved muscle contractile function ex vivo [62]. Overall, this study highlights a novel role for NUR77 in regulating oxidative metabolism and mitochondrial activity in skeletal muscle.

Nuclear factor-κB (NF-κB) is a transcription factor composed of different subunits ([p50, p52, p65 (RelA), RelB, and c-Rel]) that regulate gene expression [63,64,65]. Studies have demonstrated that endurance exercise reduces NF-κB activation, leading to beneficial effects on skeletal muscle, such as mitochondrial adaptations and prevention of protein degradation [63,64,65]. Whole-body KO mice lacking the p50 subunit of NF-κB exhibited improved long-term endurance and a lower RER during running exercise compared to wild-type mice [64]. These knockout mice also showed higher muscle glycogen levels and lower lactate production from carbohydrate metabolism after running [64]. The study suggests that the effects of p50 deficiency on fuel utilization and exercise performance may be attributed to the inhibitory action of p50 on the transcriptional activity of PPARα and PPARδ.

### 2.4. Extracellular Factors

IL-13 is a prominent Th2 cytokine that is classified as an anti-inflammatory cytokine due to its ability to suppress the secretion of several macrophage and monocyte-derived inflammatory cytokines [66]. A study showed that whole-body *Il13* KO mice demonstrated reduced running capacity on a treadmill relative to wild-type control animals [67]. Furthermore, these KO mice failed to increase the expression of mitochondrial and fatty acid oxidation genes in skeletal muscle in response to exercise. Moreover, *Il13*-KO mice had muscle that showed defective fatty acid utilization after a single bout of exercise and failed to increase mitochondrial biogenesis after endurance training. Mechanistically, it was shown that IL-13 is required to mediate active phosphorylation of STAT3, which is known to be important for performing full capacity endurance exercise. It was also found that IL13-STAT3 signaling activates several mitochondrial genes through ERRɑ and ERRg, which are orphan receptors known to regulate fatty acid catabolism and mitochondrial respiration in muscle. This study highlights the important role of IL-13 in energy substrate utilization during endurance exercise.

## 3. Glucose Utilization 

### 3.1. Factors Regulating Substrate Availability

Long-chain acyl-CoA synthetases (ACSLs) convert long-chain fatty acids to fatty acyl-CoAs for oxidation. ACSL1, found in highly oxidative tissues like the outer mitochondrial membrane, facilitates the entry of fatty acids into mitochondria for β-oxidation [68]. Muscle-specific KO of *Acsl1* (*Acsl1*M^−/−^) in mice leads to reduced β-oxidation in muscles, resulting in a significant decrease in endurance exercise capacity [69]. Although glycogen and triacylglycerol stores remain similar, *Acsl1*M^−/−^ mice exhibit lower plasma glucose concentrations and a higher RER [69], indicating an increased reliance on glucose as a fuel source. This shift towards glucose usage may be attributed to the depletion of glucose reserves caused by excessive glucose utilization and potential utilization of amino acids as fuel within the muscle, limiting substrate availability for hepatic gluconeogenesis [69]. Thus, impaired conversion of long-chain acyl-CoA to fatty acyl-CoAs and their transport into the mitochondrial matrix in skeletal muscle contributes to excessive glucose utilization.

Glycogen synthase (GYS) is an essential enzyme for glycogen synthesis in skeletal muscle [70]. There are two isoforms of GYS in mammals: GYS2, primarily expressed in the liver, and GYS1, expressed in skeletal and cardiac muscle, as well as other tissues [71]. A study utilizing muscle-specific *Gys1* KO mice (*Gys1*-KO) revealed the significant role of GYS1 in muscle glycogen synthesis and non-oxidative glucose metabolism. The *Gys1*-KO mice exhibited peripheral insulin resistance, glucose intolerance, and impaired muscle function during contraction and exercise, which affected their endurance capacity [72]. The lack of *Gys1* led to muscle glycogen depletion, resulting in reduced muscle performance and fatigue during exercise [72]. Additionally, the *Gys1*-KO mice showed decreased glucose turnover, impaired muscle glucose uptake, elevated plasma and muscle lactate levels, and reduced levels of muscle hexokinase II [72], indicating peripheral insulin resistance. These findings highlight the critical role of GYS1 in maintaining muscle glycogen levels, muscle function, and glucose metabolism during exercise.

Apoptosis-inducing mitochondrion-associated factor 2 (AIFM2) is an NADH/NAD+ oxidoreductase belonging to the apoptosis-inducing factor (AIF) protein family [73]. AIFM2 expression is significantly increased in glycolytic fast-twitch muscles during high-intensity exercise [74]. Studies using muscle-specific knockdown (KD) or haploinsufficiency of *Aifm2* in mice demonstrated reduced exercise capacity accompanied by elevated plasma glucose levels and decreased plasma free fatty acid levels [74], indicating impaired glucose utilization. These mice also exhibited a lower NAD+/NADH ratio after exercise [74]. Conversely, overexpression of AIFM2 in the fast-twitch muscles resulted in improved exercise capacity, enhanced glucose utilization, decreased fatty acid oxidation, and an increased NAD+/NADH ratio after exercise [74]. Delivery of NDE1, an external NADH oxidoreductase, reversed the exercise defects in muscle-specific *Aifm2* haploinsufficient mice by restoring the NAD+ ratio, increasing exercise capacity by approximately 30% and normalizing glucose and fatty acid utilization [74]. These findings highlight the physiological role of AIFM2 in promoting glycolysis during high-intensity exercise by facilitating NAD+ availability through its NADH oxidoreductase function.

### 3.2. Signaling Factors

S6K1, a component of the mTORC1 signaling pathway, regulates cell growth and muscle mass [75]. Resistance training activates mTORC1, leading to increased S6K1 phosphorylation and muscle hypertrophy. *S6k1* KO mice are protected against insulin resistance and glucose intolerance [75]. After exercise training, *S6k1* KO mice exhibit enhanced running capacity, reduced triglyceride levels in the liver and muscle, and increased ketogenesis [75]. This suggests improved lipid utilization and preservation of glycogen reserves. The absence of S6K1 mimics the effects of chronic endurance exercise, promoting efficient substrate utilization and carbohydrate conservation during high-fat diet consumption.

mTORC1 activity is regulated by interactions with GTPases, including RHEB and RAGs, which enable mTORC1 to localize to the lysosome for its kinase activity [76]. GATOR1, composed of NPRL2, NPRL3, and DEPDC5, acts as a negative regulator of RAG and prevents mTORC1 translocation to the lysosome during amino acid limitation [77]. Muscle-specific *Nprl2* KO mice exhibit hyperactive mTORC1 signaling, leading to an increase in glycolytic fibers and a higher RER during exercise [76]. These mice also show decreased expression of *Pgc1α*, a regulator of mitochondrial biogenesis, and increased expression of glucose uptake-related genes [76]. The activation of mTORC1 promotes mitochondrial amino acid synthesis, resulting in increased glycolysis to meet ATP demands. Similarly, muscle-specific *Depdc5* KO mice demonstrate constitutive mTORC1 activation and a tendency towards increased glycolytic muscle fibers [77]. These mice exhibit heightened mitochondrial respiratory capacity without improved endurance, indicating hyperactive mitochondria in glycolytic muscles [77]. Overall, the GATOR1 complex and mTORC1 play crucial roles in regulating carbohydrate utilization and maintaining mitochondrial homeostasis in skeletal muscle. Simultaneous activation of both RHEB and RAG via muscle-specific Tsc1/Depdc5-double knockout can lead to early-onset myopathy associated with excessive mitochondrial oxidative stress. They also showed cardiovascular defects and early fatality, further emphasizing the role of mTORC1 in mediating mitochondrial function and maintaining homeostasis [78]. 

AMPK is a kinase involved in energy metabolism and gene transcription, acting as a whole-body stress sensor [79,80]. It is activated in response to energy-depleting conditions and integrates signals from exercise, nutrients, and hormones to regulate food intake, energy expenditure, and substrate usage [81]. AMPK consists of α, β, and γ subunits, with the β subunits playing a critical role in assembling the heterotrimer and regulating enzyme activity and localization [80]. In skeletal muscle, α2β2γ3-containing heterotrimers are predominant. KO mice lacking β1 and β2 subunits (β1β2m-KO) show reduced physical activity and impaired treadmill running capacity, accompanied by decreased skeletal muscle mitochondrial content [80]. Notably, β1β2m-KO mice fed a normal diet do not exhibit changes in body weight or insulin sensitivity but display impaired glucose uptake during muscle contractions, indicating the importance of AMPK in glucose utilization [80]. Further research is needed to fully understand the precise role of AMPK in skeletal muscle.

### 3.3. Transcription Factors

PPARδ is a nuclear receptor that plays a crucial role in regulating fatty acid metabolism in muscle. PPARδ overexpression in skeletal muscle promotes the transformation of muscle fibers to an oxidative type and enhances fatty acid oxidation by increasing the expression of two important mitochondrial proteins: carnitine palmitoyl-transferase 1b and pyruvate dehydrogenase kinase isozyme 4 [82]. This metabolic adaptation is dependent on muscle PPARδ and can be stimulated by PPARδ ligands [82]. Importantly, higher expression of muscle PPARδ is associated with improved endurance performance. Additionally, PPARδ activation suppresses glucose breakdown without affecting muscle fiber type or mitochondrial content [82]. By preserving glucose levels, PPARδ helps to delay the onset of low blood sugar and significantly prolong running time in treated mice [82]. These findings highlight the dual role of PPARδ in promoting glucose sparing and suggest the potential use of PPARδ-targeted exercise mimetics in treating metabolic disorders, muscular dystrophies, and potentially enhancing athletic performance.

The myogenic transcription factor Myogenin is crucial for the development of skeletal muscle during embryonic and fetal stages [83]. Surprisingly, inducible whole-body *Myog* KO mice at 12 weeks of age exhibited normal muscle development but showed improved performance compared to controls in both high- and low-intensity treadmill running [83]. This enhanced exercise capacity was attributed to more efficient oxidative metabolism during low- and high-intensity exercise, as well as more efficient glycolytic metabolism during high-intensity exercise [83]. Notably, *Myog* deletion resulted in excessive depletion of blood glucose during intense exercise [83]. Additionally, *Myog* KO mice displayed higher trainability in voluntary exercise training on running wheels [83], suggesting that Myogenin plays a critical role in regulating the balance between aerobic and anaerobic metabolism in adult skeletal muscle.

Hypoxia-Inducible Factor (HIF)-1α plays an integral role in the body’s response to low oxygen concentrations, or hypoxia [84]. During exercise, hypoxia influences important aspects of muscle function, including ATP production, energy substrate utilization, and the generation of metabolites that contribute to fatigue [85]. Glycolysis, the anaerobic energy pathway, is regulated by HIF-1a under conditions of low oxygen availability [85]. Muscle-specific *Hif1a* KO mice exhibited a shift in fuel utilization from glucose to lipid oxidation. They demonstrated resistance to the exercise-induced activation of genes involved in glycolysis and glucose uptake, such as *Pfkm*, *Glut4*, *Pgk*, and *Ldha* [85]. During exercise, the KO mice had reduced levels of serum lactate, indicating decreased reliance on glycolysis [86] while mitochondrial activity increased [85]. However, repeated exercise led to shorter exercise times in the knockout mice compared to the control group, primarily due to extensive muscle damage [85]. Additionally, the KO mice displayed severe impairments in skeletal muscle glycogenolysis and glycolysis [85]. Overall, this study highlights the role of HIF-1a in promoting glucose utilization over lipid utilization during exercise. 

### 3.4. Extracellular Factors

IL-6 was the first myokine discovered to be released into the bloodstream in response to muscle contractions [87] and is one of the most extensively studied exerkines [88]. Its concentration can increase up to 100-fold during exercise. Studies using whole-body *Il6* KO mice have shown that these mice have reduced endurance capacity compared to wild-type mice [89]. This reduced endurance is accompanied by significant intramuscular glycogen depletion and increased expression of glucose transporters (GLUT1, GLUT4), without changes in fatty acid transporters [89]. *Il6* KO mice also exhibit shorter swim durations compared to WT mice [89]. IL-6 has been found to regulate glucose and fatty acid uptake through the upregulation of AMPK, an important regulator of fatty acid oxidation [89]. Decreased AMPK activation in *Il6* KO mice during exercise leads to reduced fatty acid flux and oxidation, resulting in increased expression of *Glut4* and increased glucose utilization [89]. Additional studies on *Il6* KO mice have demonstrated impaired exercise capacity at high velocities and significantly lower plasma lactate levels after exercise, indicating impaired anaerobic glycolysis and increased glycogen depletion [90]. Muscle-specific *Il6* KO mice also exhibit higher RER levels during exercise, indicating a preference for carbohydrate use over lipid use, which is accompanied by increased pyruvate dehydrogenase (PDH) activity [91]. These muscle-specific *Il6* KO mice also had lower liver glucose level than WT mice during prolonged exercise, which may explain the higher RER and increased PDH activity, and indicates that skeletal muscle IL-6 may indirectly stimulate hepatic glucose production during exercise [92]. Administration of IL-6 recombinant to mice has been shown to increase exercise endurance and the expression of genes involved in oxidative phosphorylation, including pyruvate dehydrogenase kinase isozyme 4, which inhibits PDH activity [87]. Human studies have supported these findings by showing that injection of IL-6 recombinant into skeletal muscle increases systemic fatty acid oxidation [93]. Together, these studies indicate that IL-6 plays a critical role in balancing the utilization of fatty acids and glycogen during endurance exercise.

## 4. Conclusions

The regulation of fuel utilization in skeletal muscle is a complex interplay of multiple mechanisms, encompassing substrate bioavailability, transport, mitochondrial fuel utilization, transcriptional control, extracellular signaling, and metabolic shifts between glucose and lipid metabolism. This intricacy underscores the multifaceted nature of fuel utilization regulation. Furthermore, it is crucial to acknowledge that fuel utilization within skeletal muscle is not isolated but instead interacts with other tissues and organ systems, such as the liver and adipose tissue. 

Moreover, understanding fuel utilization during exercise requires consideration of the broader context. Factors like pre-exercise metabolism influenced by nutrition and post-exercise factors like exercise type, intensity, duration, and environmental conditions all play integral roles and must be examined for a holistic understanding. Additionally, investigating the impact of fiber type heterogeneity, sex, and factors like menstrual cycles on metabolic rate and energy substrate partitioning is essential. These variables reflect the complexity of exercise metabolism.

Skeletal muscle, a major contributor to metabolic rate and energy utilization, assumes a central role in glycemic control, impacting glucose homeostasis under both insulin-stimulated and independent conditions. During exercise, skeletal muscle dynamically adjusts energy production, blood flow, and substrate utilization, influenced by factors like exercise intensity, duration, and hormonal responses, affecting the balance between carbohydrates and lipids as energy sources. After exercise, there is a significant shift toward lipid oxidation during the recovery period, vital for muscle glycogen replenishment. Understanding these metabolic intricacies is crucial for comprehending short- and long-term responses to exercise.

Overall, the metabolic conditions of the whole organism and the interactions between different tissues significantly shape fuel utilization in skeletal muscle. The differences between muscle-specific and whole-body knockout studies underscore the importance of the interplay between different tissues in regulating fuel utilization. To achieve a comprehensive understanding, future research should delve into the specific functions of various factors and mechanisms in a tissue-specific manner while considering interactions between tissues. This deeper understanding of fuel utilization can provide valuable insights into how exercise and skeletal muscle contribute to human health and overall quality of life. A summary of the results are presented in Table 1.

## Figures and Tables

**Table 1:** Summary of Factors Affecting Fuel Utilization in Skeletal Muscle During Exercise.

Gene/Protein	Genetic Manipulation	Associated Function	Exercise Phenotype	Effects on Fuel Utilization	References
CD36	Whole-body KO	Fatty acid transporter	Promote endurance exercise capacity	Promote fatty acid transport into muscle, allowing for metabolic switching	[14]
ATGL	Whole-body KO	Triacylglycerol lipase	Promote endurance exercise capacity	Promote exercise-stimulated lipolysis	[19]
HSL	Whole-body KO	Triacylglycerol lipase	Promote endurance exercise capacity	Promote exercise-stimulated lipolysis	[20]
L-Carnitine	Overexpression (oral dose administered)	Cofactor for fatty acid transformation and transport	Enhance endurance exercise capacity	Promote fat utilization and mitochondrial biogenesis	[23]
VEGF	Adipocyte-specific KO	Promotes angiogenesis	Promote exercise tolerance	Increase adipose capillarity, increasing bioavailability of free fatty acids	[25]
OPA1	Haplodeficiency	Regulate mitochondrial fusion	Not sufficient to affect exercise capacity by itself	Promote fatty acid oxidation	[30]
NRF2	Whole-body KO	Regulate genes in oxidative stress response	Promote endurance exercise capacity	Promote fatty acid oxidation	[33,34]
KEAP1	Muscle-specific KO	Negatively regulates NRF2	Decrease endurance and resistance exercise capacity	Decreases fatty acid oxidation	[32]
PGC-1a	Whole-body KO, Muscle-specific KO	Cotranscription factor that regulates fatty acid oxidation, mitochondrial biogenesis, and mitochondrial function	Promote exercise tolerance and slow-twitch muscle fiber content	Maintain glucose homeostasis, promote fatty acid oxidation	[35,37,38,39]
Sestrins	Whole-body KO	Stress-induced protein, activates downstream pathways to regulate metabolism	Promote endurance exercise capacity	Maintain glucose homeostasis, promote fatty acid oxidation	[50]
PPARα	Whole-body KO, muscle-specific overexpression	Transcription factor that regulates lipid and glucose homeostasis	Promote endurance exercise capacity	Favor lipid utilization over glucose, promotes fatty acid oxidation	[54,57,58]
KLF15	Whole-body KO	Transcription factor that regulates many metabolic processes	Promote endurance exercise capacity	Allows metabolic switching; promotes fatty acid oxidation in endurance exercise	[60]
NR4A1 or NUR77	Muscle-specific overexpression	Transcription factor that affects muscle fiber content	Increase muscle contractile function	Promotes fatty acid oxidation over glucose oxidation	[62]
P50 (NF-κB)	Whole-body KO	Transcription factor that regulates inflammation and infection	Decrease endurance exercise capacity	Decreases fatty acid oxidation and promotes glucose oxidation	[64]
Il-13	Whole-body KO	Anti-inflammatory cytokine	Promote exercise capacity	Promote fatty acid oxidation and mitochondrial biogenesis	[67]
ACSL1	Muscle-specific KO	Directs fatty acids into mitochondria for fatty acid oxidation	Promote endurance exercise capacity	Promotes fatty acid oxidation over glucose use	[69]
GYS	Muscle-specific KO	Key enzyme in synthesis of glycogen in skeletal muscle	Promote endurance exercise capacity	Maintains muscle glycogen for use as a fuel source	[72]
AIFM2	Muscle targeted KD and OE, muscle-specific haploinsufficiency (ACTA-Cre)	NADH oxidoreductase	Promote high-intensity exercise	Promote glucose utilization over lipid use	[74]
S6K1	Whole-body KO	Component of mTORC signaling pathway that controls cell growth	Decrease exercise capacity	Downregulate ketogenesis, and promote glucose utilization over fatty acid oxidation	[75]
mTORC1	Muscle-specific activation (via KO of negative regulators)	Regulator of protein translation and cellular metabolism	Muscle hypertrophy; no observed differences in exercise capacity	Increase glycolytic fibers in muscle, promoting glucose utilization	[76,77,78]
AMPK	Whole-body KO	Whole-body stress sensing kinase that regulates energy metabolism	Promote exercise capacity	Promote mitochondrial content and glucose uptake	[80]
PPARδ	Muscle-specific overexpression	Nuclear receptor that is a key regulator of fatty acid metabolism in muscle	Promote endurance exercise capacity	Stimulate fatty acid oxidation and suppress glucose utilization	[82]
Myogenin	Whole-body KO	Regulates skeletal muscle development during embryonic and fetal stages	Decrease exercise capacity	Increase sensitivity to depletion of glycogen reserves; decreased efficiency of fatty acid and glucose use	[83]
HIF-1α	Muscle-specific KO	Protein complex that responds to low oxygen conditions (hypoxia)	Decrease exercise capacity; prevent muscle damage from repeated exercise	Promotes glucose utilization over fatty acid oxidation	[85]
Il-6	Whole-body KO, muscle-specific KO, whole-body OE (injection), muscle-specific OE (injection)	Myokine believed to be largely anti-inflammatory	Increase exercise capacity	Increase fatty acid oxidation	[87,89,90,93]

## Data Availability

No new data were created or analyzed in this study. Data sharing is not applicable to this article.
